# Supplementary material for: Conformational Properties of New Thiosemicarbazone and Thiocarbohydrazone Derivatives and Their Possible Targets
Source: Molecules. 2022 Apr 14;27(8):2537. doi: 10.3390/molecules27082537 (PMC9028911; doi:10.3390/molecules27082537)
Supplement: Supplementary file 1 [file molecules-27-02537-s001.zip › molecules-1638465-supplementary.pdf]

SUPPORTING INFORMATION

Nikitas Georgiou[1], Aikaterini Katsogiannou[1], Dimitrios Skourtis[2], Hermis Iatrou[2], Demeter Tzeli[3,4], Stamatia Vassiliou[1*], Uroš Javornik[5], Janez Plavec[5], Thomas Mavromoustakos[1*]

[1]National and Kapodistrian University of Athens, Department of Chemistry, Laboratory of Organic Chemistry, Panepistimioupolis Zografou, 11571, Athens
[2]National and Kapodistrian University of Athens, Department of Chemistry, Laboratory of Polymer Chemistry, Panepistimioupolis Zografou, 11571, Athens
[3]National and Kapodistrian University of Athens, Department of Chemistry, Laboratory of Physical Chemistry, Panepistimioupolis Zografou, 11571, Athens
[4]Theoretical and Physical Chemistry Institute, National Hellenic Research Foundation, 48 Vassileos Constantinou Ave., Athens 116 35, Greece
[5] Slovenian NMR Centre, National Institute of Chemistry, SI-1001 Ljubljana, Slovenia

***** Correspondence: svassiliou@chem.uoa.gr (S.V.); tmavrom@chem.uoa.gr (T.M.)

Table S1. Assignment of the 13C spectrum of KKI15 in DMSO-d6 (top) and KKI18 in DMSO-d6 (bottom)

Peak	Chemical shift (ppm)	Peak	Chemical shift (ppm)	Peak	Chemical shift (ppm)
13	127.39	15	131.27	9	119.18
14	128.74	10	129.37	5	140.15
11	129.69	7	137.29	2	179.40
12	129.14	7’	128.42	5’	137.09
8	136.26	6	136.13		

Peak	Chemical shift (ppm)	Peak	Chemical shift (ppm)	Peak	Chemical shift (ppm)
14	128.54	11	129.12		
16	127.43	8	139.81		
15	129.67	7	136.15		
12	130.21	10	119.08		
13	128.69	6	148.55		
9	137.25	3	176.44		

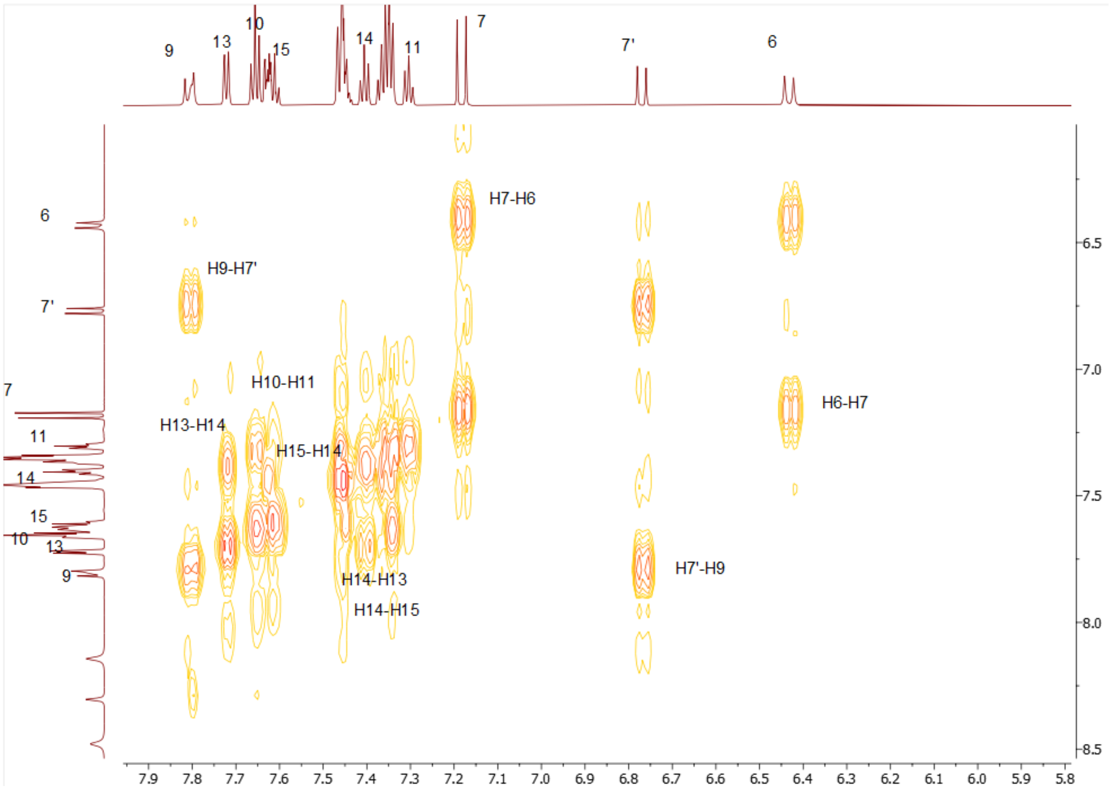

Figure S1A. 2D-COSY-NMR spectra. The spectra were recorded in DMSO-d_6 on a Bruker AC 800MHz spectrometer at 25ºC.

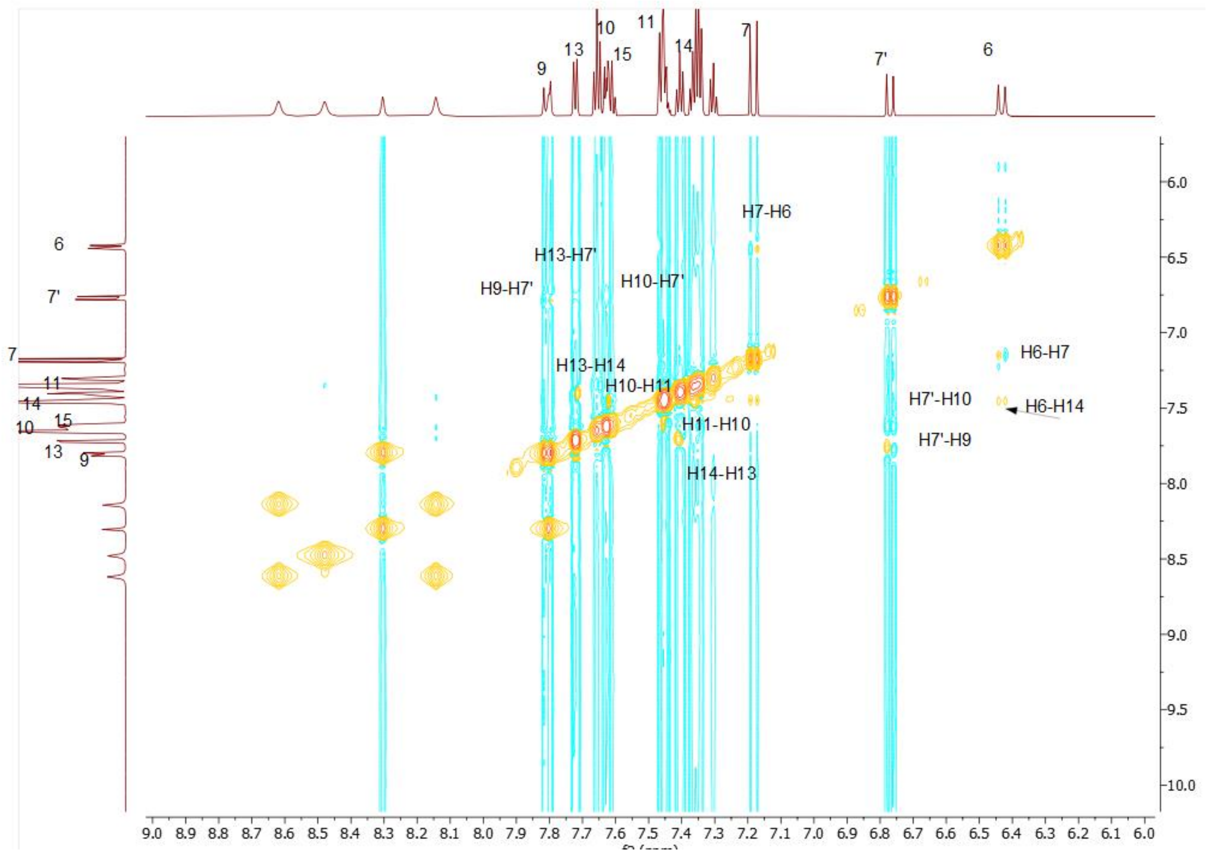

Figure S1B. 2D-NOESY-NMR spectra. The spectra were recorded in DMSO-d_6 on a Bruker AC 800MHz spectrometer at 25°C.

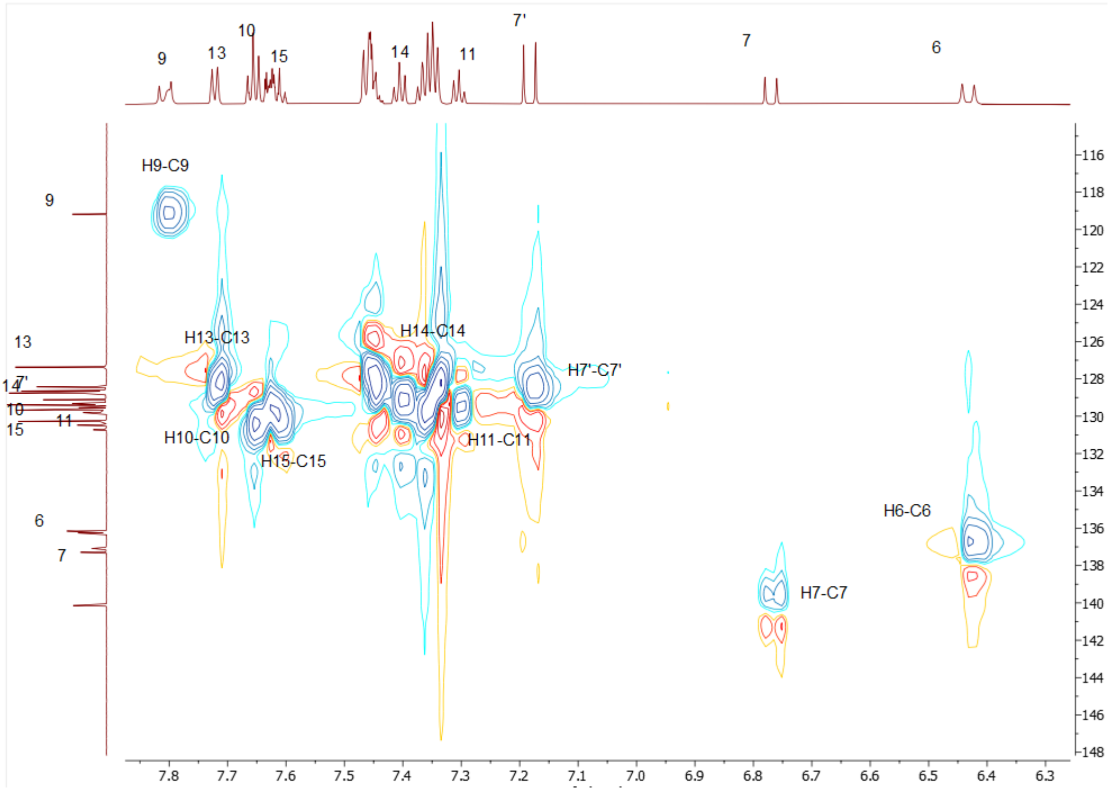

Figure S1C. 2D-HSQC-NMR spectra. The spectra were recorded in DMSO-d_6 on a Bruker AC 800MHz spectrometer at 25°C.

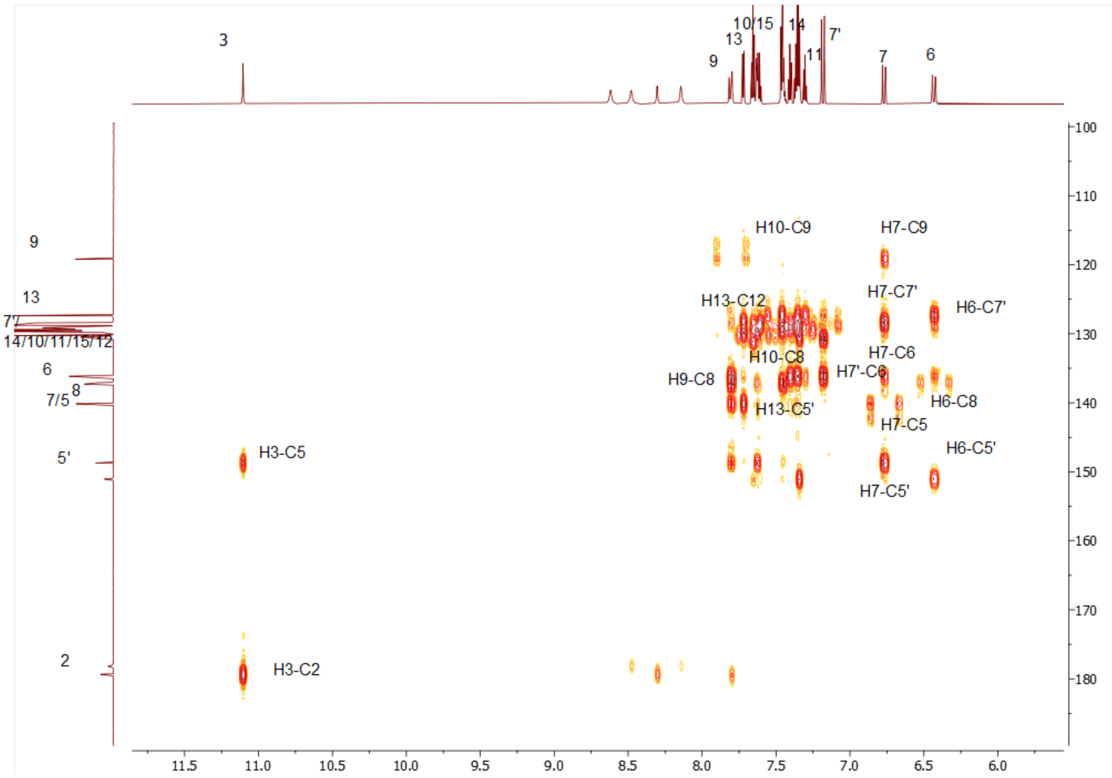

Figure S1D. 2D-HMBC-NMR spectra. The spectra were recorded in DMSO-d_6 on a Bruker AC 800MHz spectrometer at 25°C.

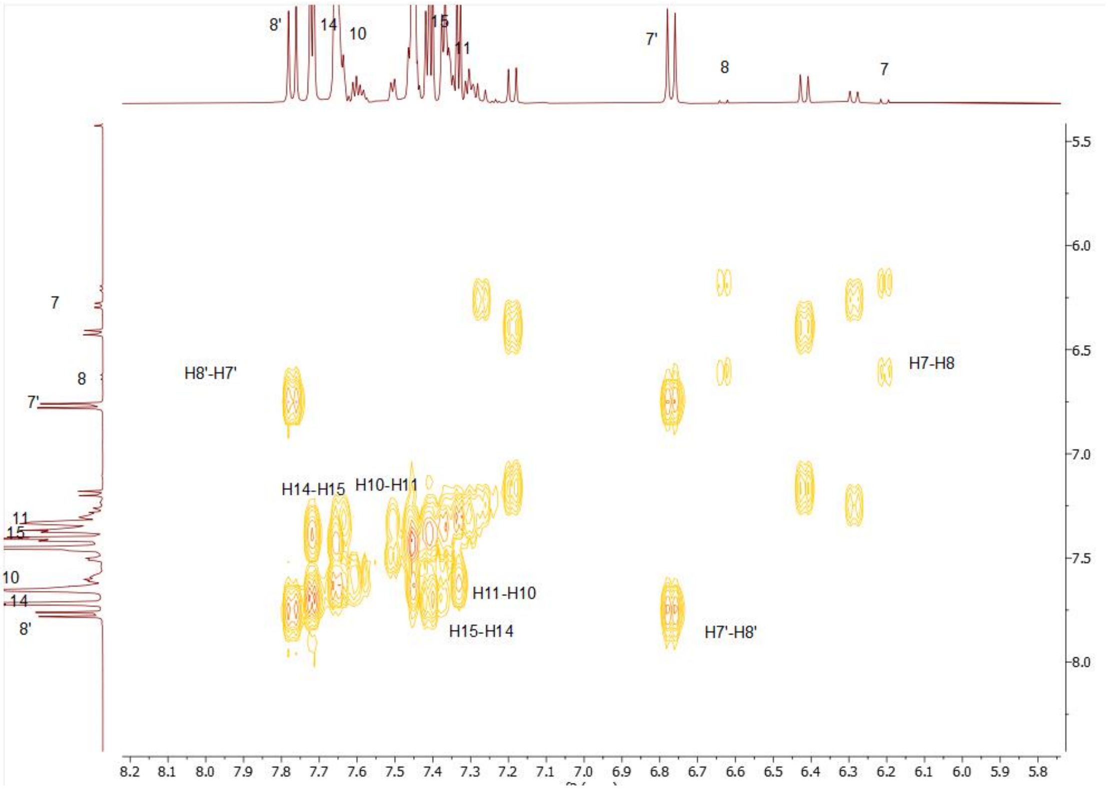

Figure S2A. 2D-COSY-NMR spectra. The spectra were recorded in DMSO-d_6 on a Bruker AC 800MHz spectrometer at 25°C.

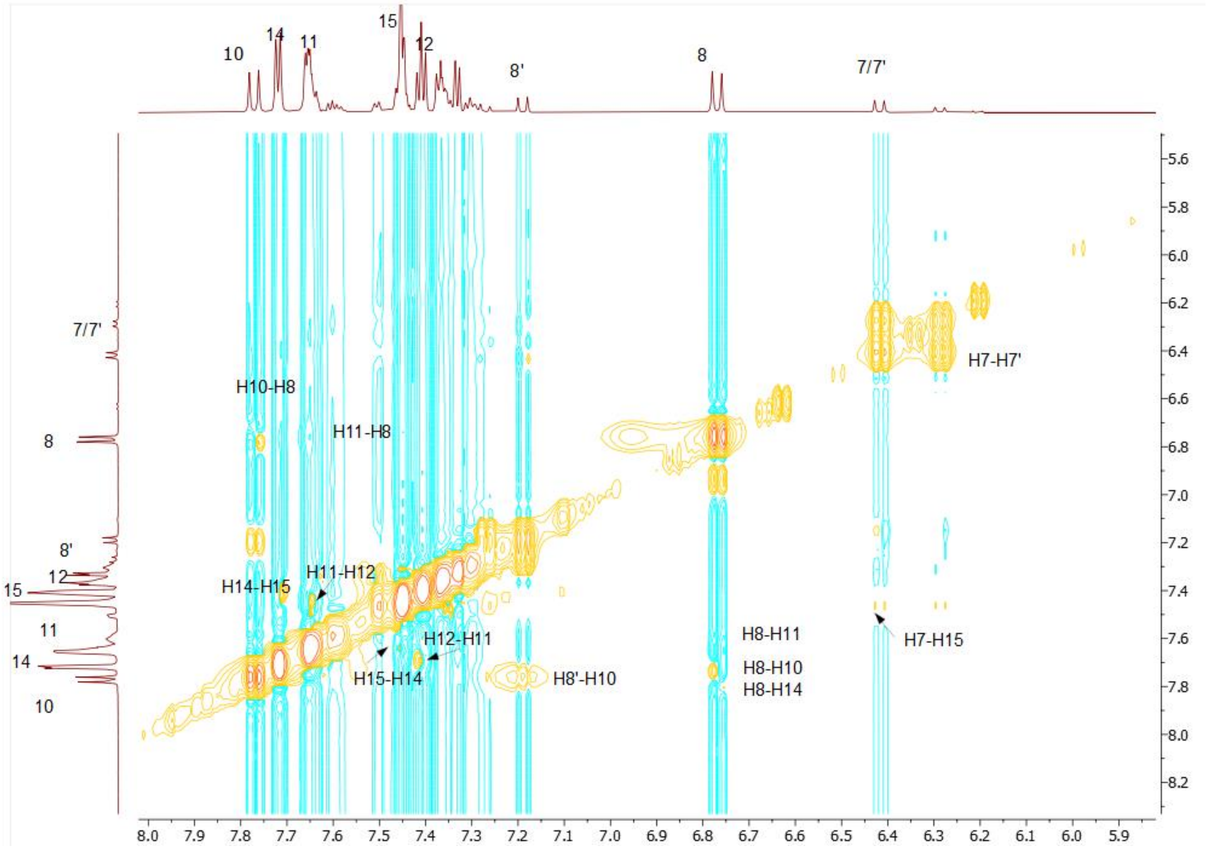

Figure S2B. 2D-NOESY-NMR spectra. The spectra were recorded in DMSO-d_6 on a Bruker AC 800MHz spectrometer at 25°C.

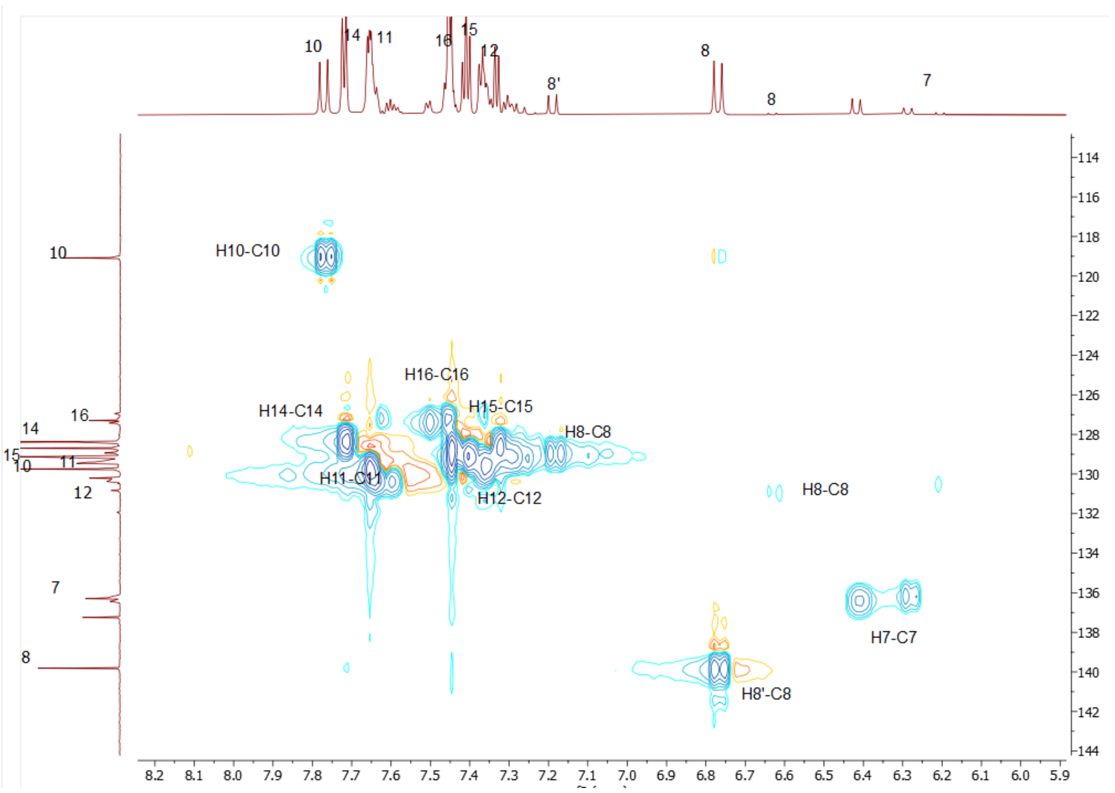

Figure S2C. 2D-HSQC-NMR spectra. The spectra were recorded in DMSO-d_6 on a Bruker AC 800MHz spectrometer at 25°C.

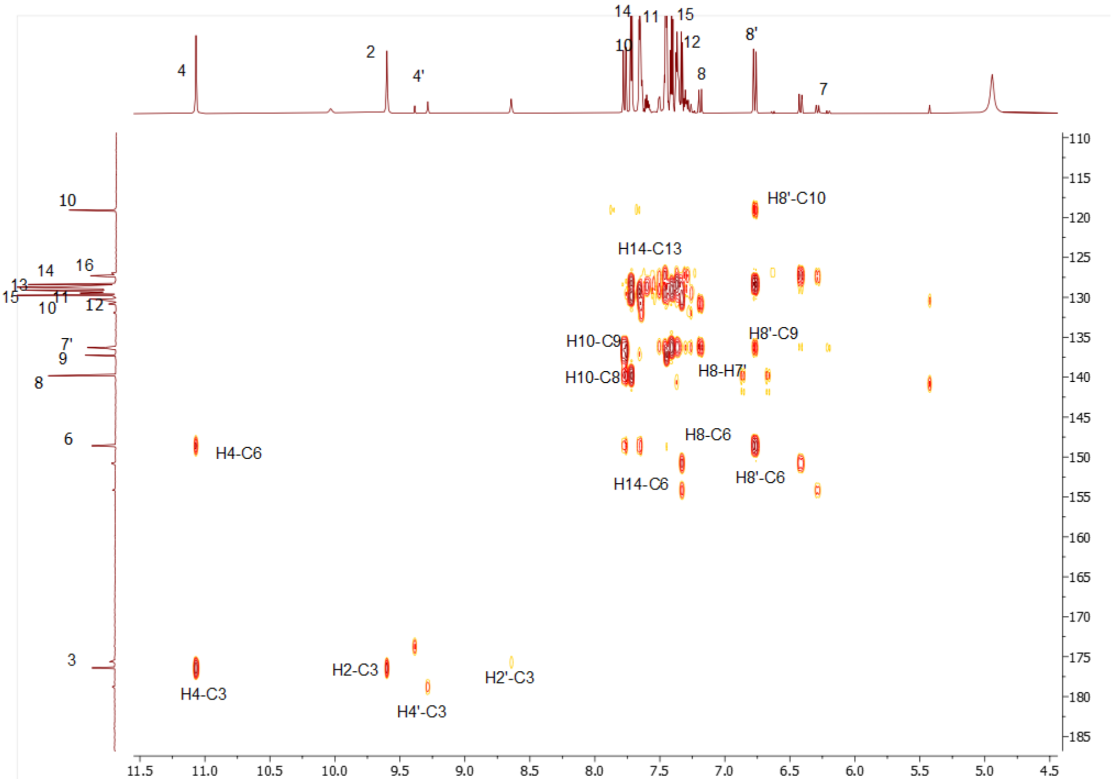

Figure S2D. 2D-HMBC-NMR spectra. The spectra were recorded in DMSO-d_6 on a Bruker AC 800MHz spectrometer at 25^oC.

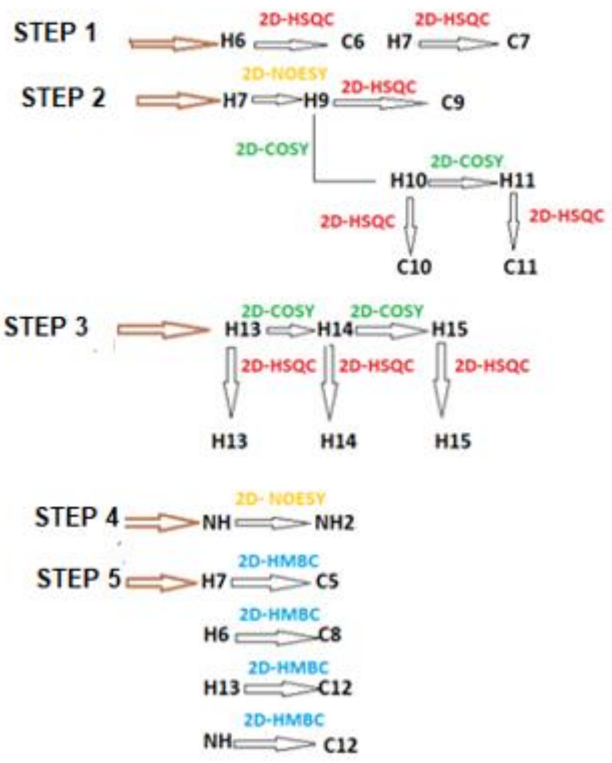

Figure S3A. Overall diagram showing the identification strategy of theKKI15 compound in DMSO.

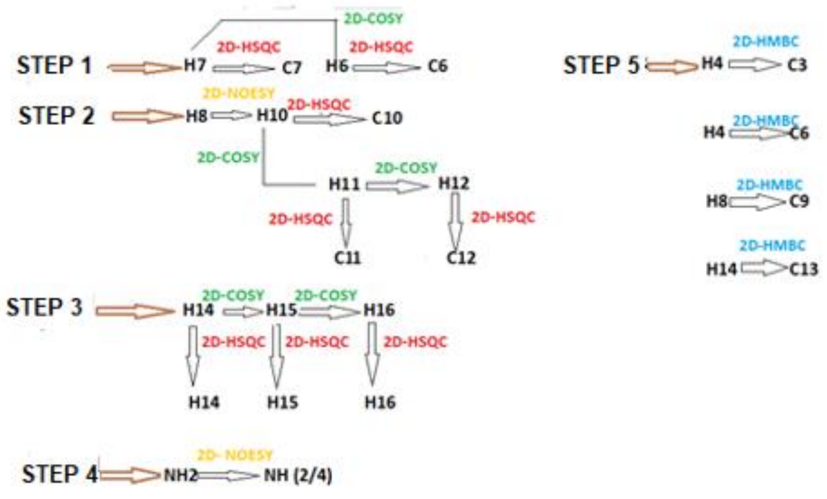

Figure S3B. Overall diagram showing the identification strategy of the KKI18 compound in DMSO.

Table S2A. Charges calculations results for compound KKI18

KKI18	H(27)								
	b3lyp/6-311G(d,p)	b3lyp/6-311+G(d,p)	b3lyp/6-311++G(d,p)	M062X/6-311G(d,p)	M062X/6-311+G(d,p)	wB97xd/6-311G(d,p)	M062X/6-311++G(d,p)	wB97xd/6-311+G(d,p)	wB97xd/6-311++G(d,p)
Mull	0.128	0.144	0.186	0.170	0.171	0.168	0.199	0.168	0.214
NBO	0.208	0.213	0.210	0.216	0.219	0.218	0.217	0.218	0.215
CM5	0.102	0.102	0.102	0.104	0.104	0.105	0.105	0.105	0.105
KKI18	H(28)								
	b3lyp/6-311G(d,p)	b3lyp/6-311+G(d,p)	b3lyp/6-311++G(d,p)						
Mull	0.121	0.144	0.244	0.163	0.178	0.172	0.26	0.172	0.276
NBO	0.203	0.205	0.205	0.209	0.212	0.211	0.211	0.211	0.209
CM5	0.104	0.103	0.104	0.106	0.106	0.107	0.106	0.107	0.107
KKI18	C(8)								
	b3lyp/6-311G(d,p)	b3lyp/6-311+G(d,p)	b3lyp/6-311++G(d,p)						
Mull	-0.195	0.172	0.082	-0.239	0.279	0.162	0.138	0.162	0.037
NBO	-0.248	-0.240	-0.246	-0.256	-0.249	-0.249	-0.255	-0.249	-0.254
CM5	0.012	0.012	0.012	0.015	0.013	0.013	0.013	0.013	0.013
KKI18	C(9)								
	b3lyp/6-311G(d,p)	b3lyp/6-311+G(d,p)	b3lyp/6-311++G(d,p)						
Mull	-0.076	-0.339	-0.496	-0.108	-0.421	-0.363	-0.478	-0.363	-0.532
NBO	-0.174	-0.172	-0.156	-0.175	-0.172	-0.170	-0.158	-0.17	-0.156
CM5	0.023	0.023	0.024	0.026	0.026	0.027	0.026	0.027	0.027

Table S2B. Charges calculations results for compound KKI15

KKI15	H(27)								
	b3lyp/6-311G(d,p)	b3lyp/6-311+G(d,p)	b3lyp/6-311++G(d,p)	M062X/6-311G(d,p)	M062X/6-311+G(d,p)	wB97xd/6-311G(d,p)	M062X/6-311++G(d,p)	wB97xd/6-311+G(d,p)	wB97xd/6-311++G(d,p)
Mull	0.122	0.146	0.255	0.165	0.180	0.173	0.273	0.173	0.289
NBO	0.203	0.207	0.205	0.209	0.213	0.211	0.211	0.211	0.208
CM5	0.105	0.105	0.105	0.107	0.107	0.108	0.107	0.108	0.108
KKI15	H(26)								
	b3lyp/6-311G(d,p)	b3lyp/6-311+G(d,p)	b3lyp/6-311++G(d,p)						
Mull	0.129	0.147	0.187	0.171	0.174	0.171	0.201	0.171	0.216
NBO	0.209	0.214	0.212	0.216	0.221	0.219	0.218	0.219	0.216
CM5	0.103	0.103	0.103	0.105	0.105	0.106	0.105	0.106	0.106
KKI15	C(8)								
	b3lyp/6-311G(d,p)	b3lyp/6-311+G(d,p)	b3lyp/6-311++G(d,p)						
Mull	-0.198	0.097	0.111	-0.241	0.187	0.088	0.167	0.088	0.079
NBO	-0.249	-0.243	-0.249	-0.258	-0.252	-0.252	-0.258	-0.252	-0.256
CM5	0.013	0.013	0.013	0.014	0.014	0.014	0.014	0.014	0.014
KKI15	C(9)								
	b3lyp/6-311G(d,p)	b3lyp/6-311+G(d,p)	b3lyp/6-311++G(d,p)						
Mull	-0.077	-0.317	-0.462	-0.109	-0.401	-0.351	-0.449	-0.351	-0.507
NBO	-0.175	-0.171	-0.156	-0.175	-0.172	-0.171	-0.158	-0.171	-0.136
CM5	0.025	0.025	0.025	0.026	0.027	0.028	0.027	0.028	0.028

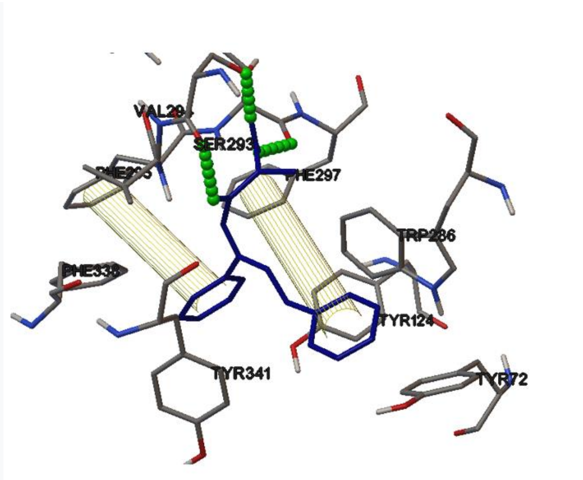

(a)

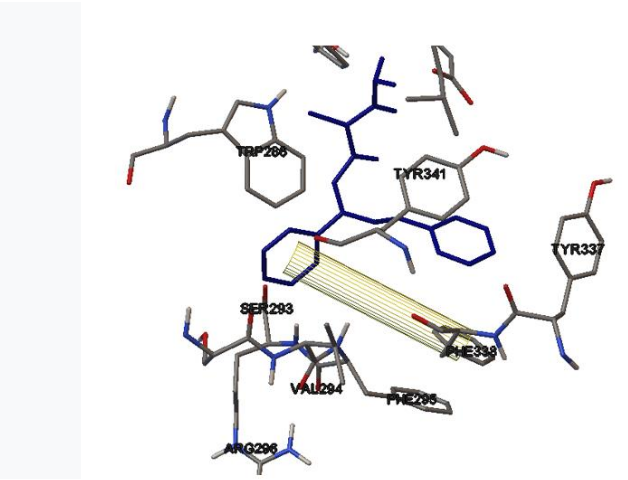

(b)

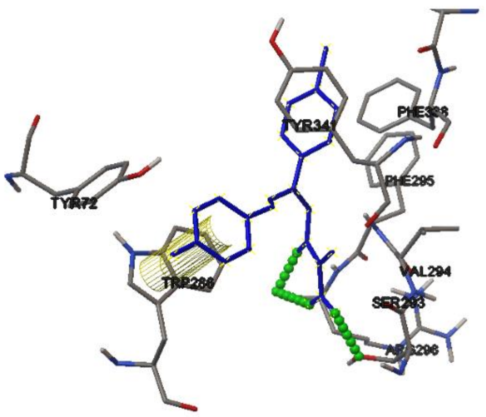

(c)

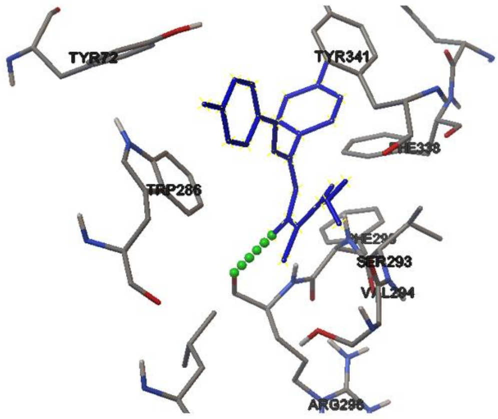

(d)

Figure S4: Binding mode of KKI15(a), KKI18(b), test_1 (c) and test_2 with acetylcholinesterase, which gave the most favorable results.

Calculated absorption and ECD spectra for compounds KKI15 and KKI18

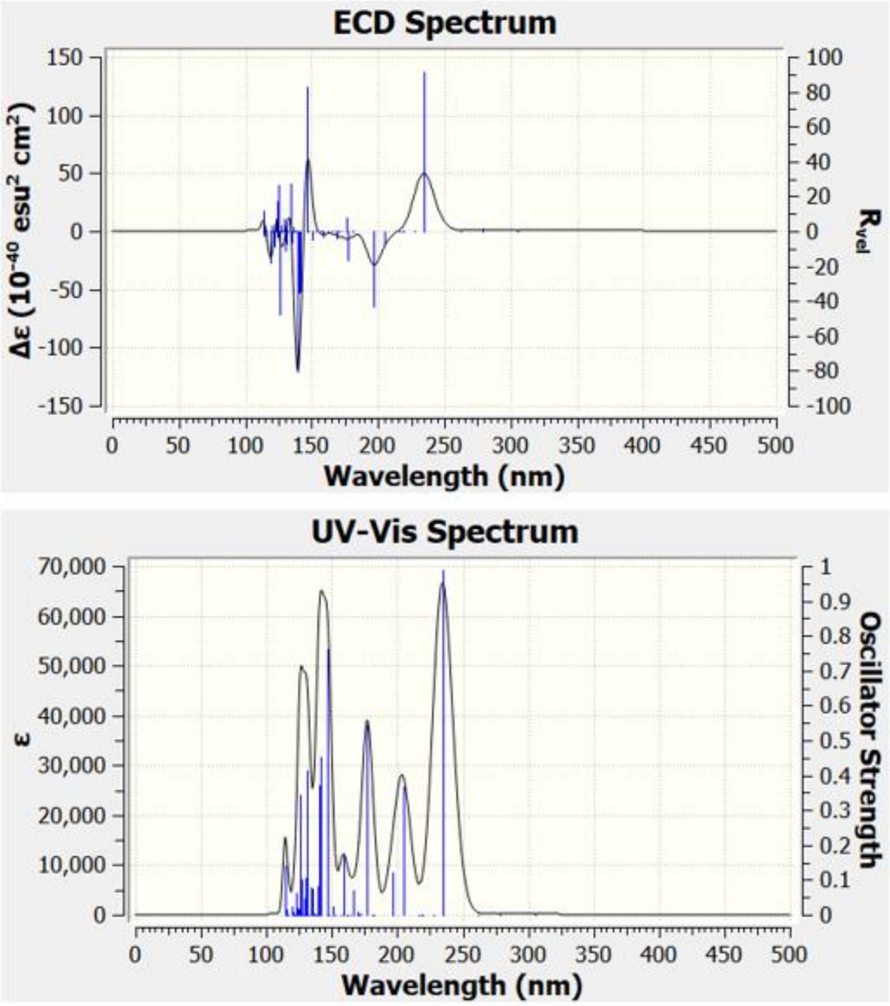

Figure S5: Theoretical extinctions (bottom) and ECD spectrum (top) of KKI15 (*E*,*E*) calculated at the B3LYP/6-311+G(d,p) level of theory.

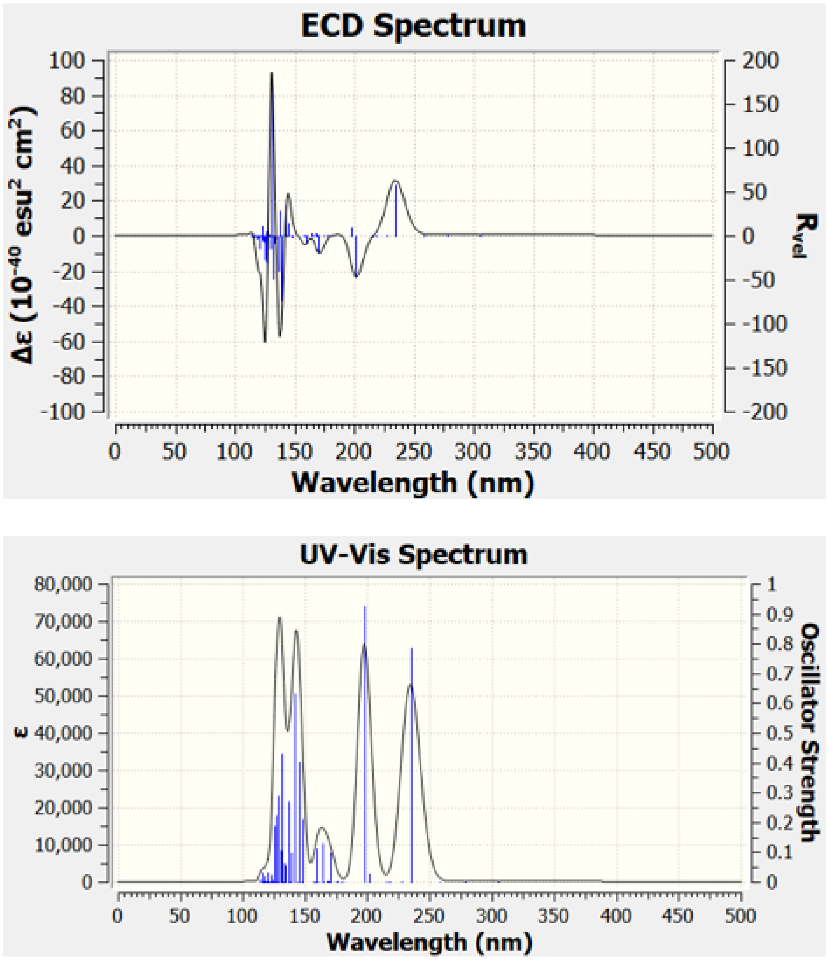

Figure S6: Theoretical extinctions (bottom) and ECD spectrum (top) of KKI15 (*E,Z*) calculated at the B3LYP/6-311+G(d,p) level of theory.

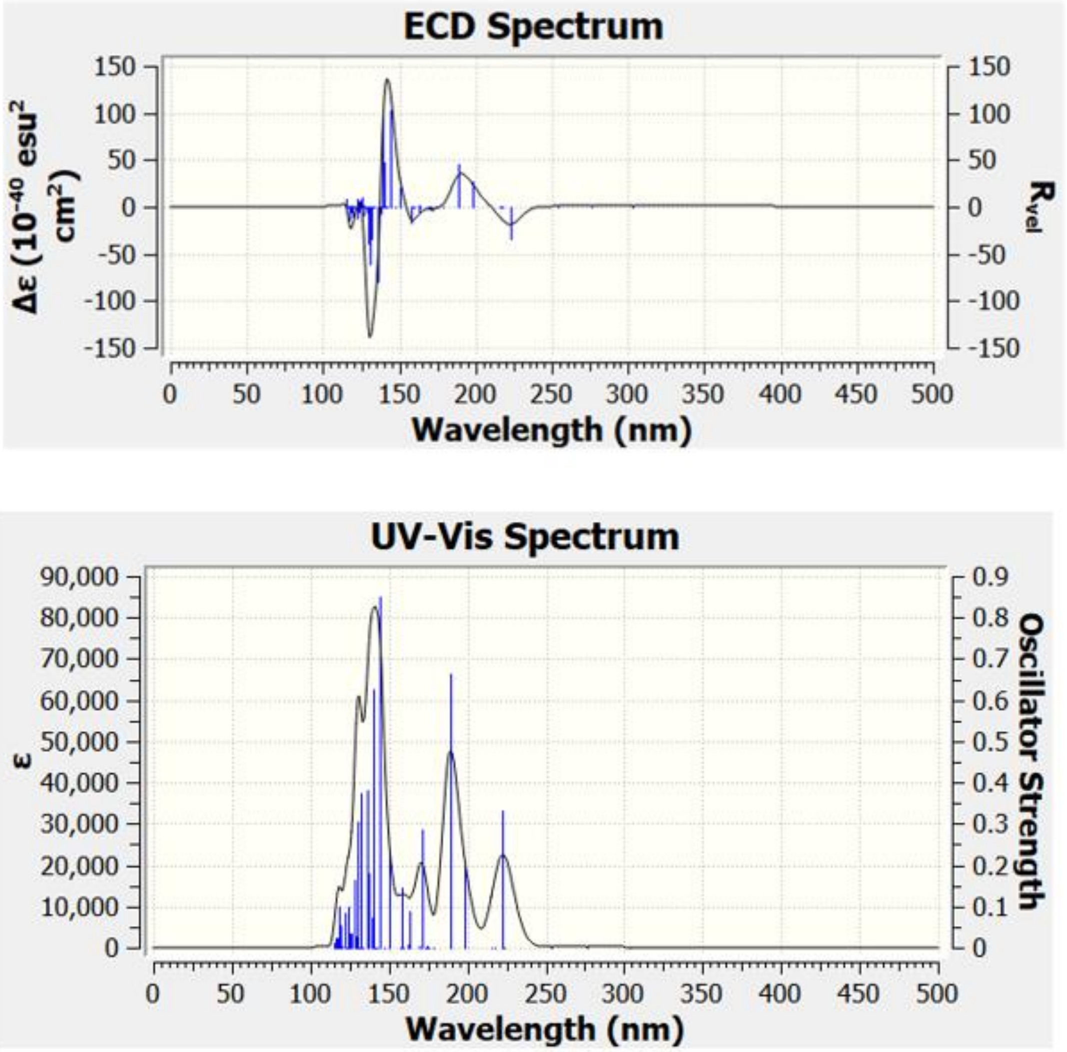

Figure S7: Theoretical extinctions (bottom) and ECD spectrum (top) of KKI15 (*Z,Z*) calculated at the B3LYP/6-311+G(d,p) level of theory.

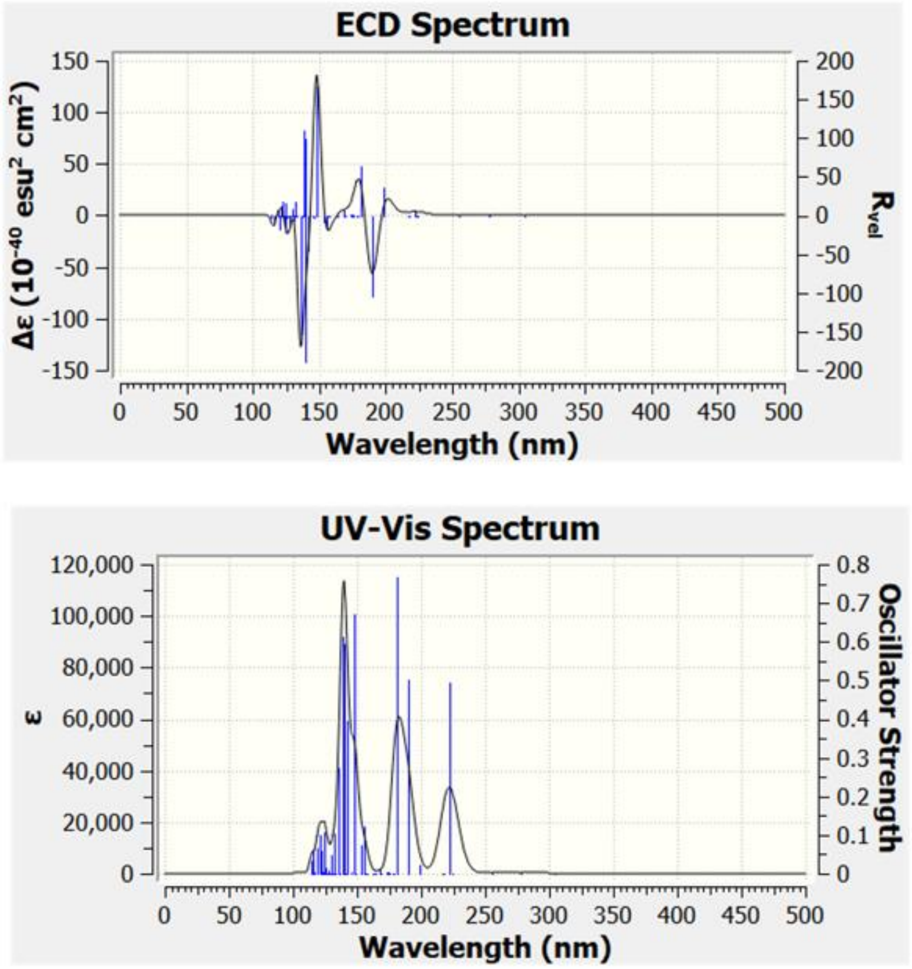

Figure S8: Theoretical extinctions (bottom) and ECD spectrum (top) of KKI15 (*Z,E*) calculated at the B3LYP/6-311+G(d,p) level of theory.

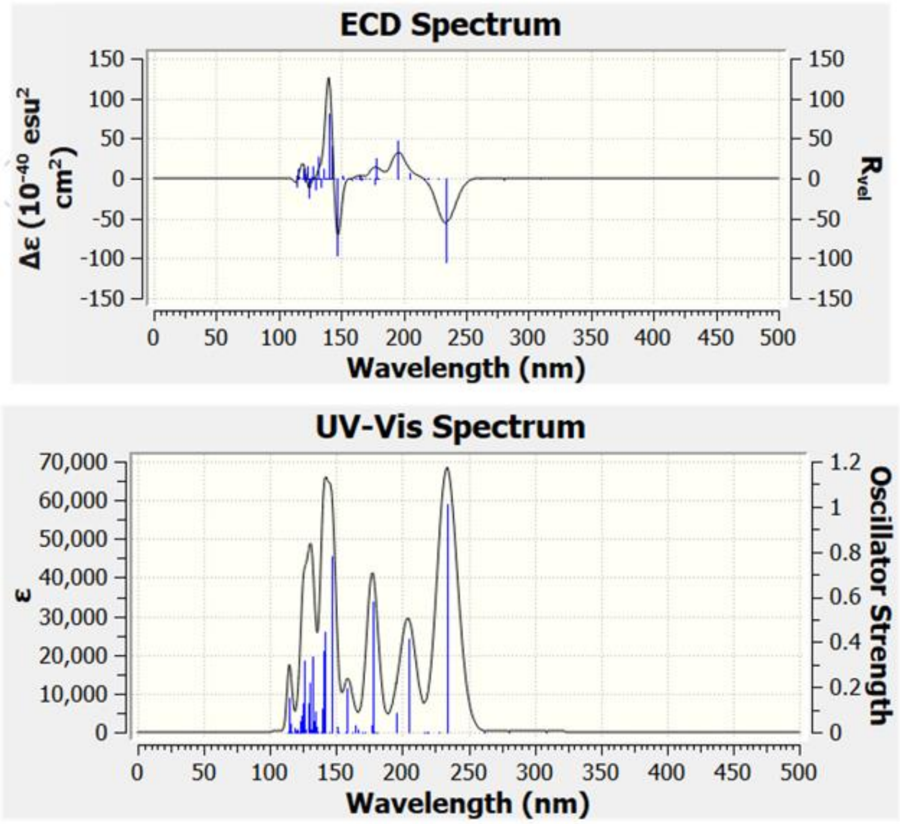

Figure S9: Theoretical extinctions (bottom) and ECD spectrum (top) of KKI18 (*E,E*) calculated at the B3LYP/6-311+G(d,p) level of theory.

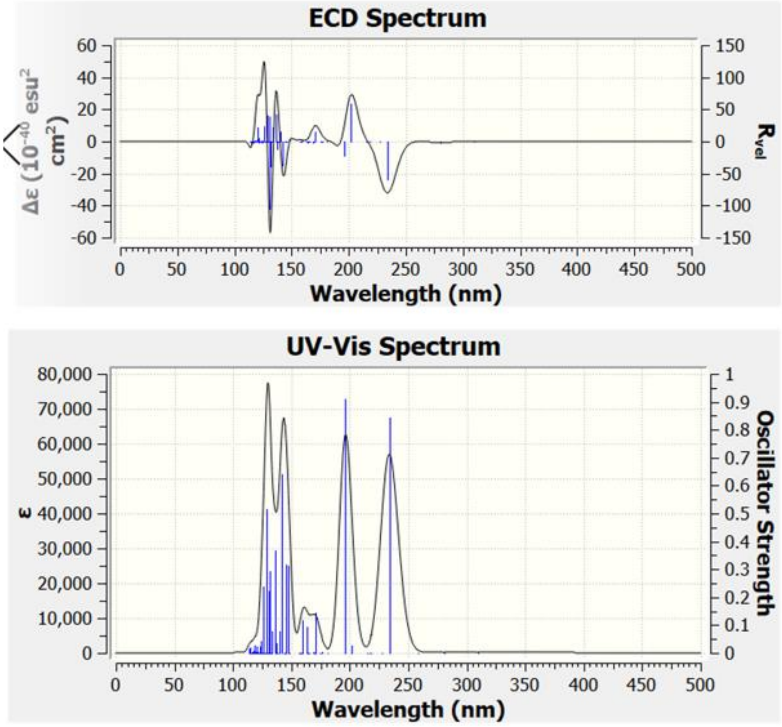

Figure S10: Theoretical extinctions (bottom) and ECD spectrum (top) of KKI18 (*E,Z*) calculated at the B3LYP/6-311+G(d,p) level of theory.

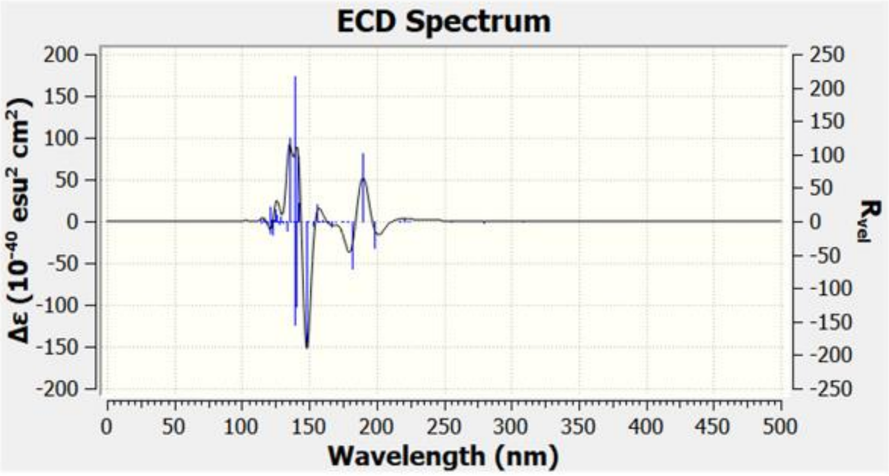

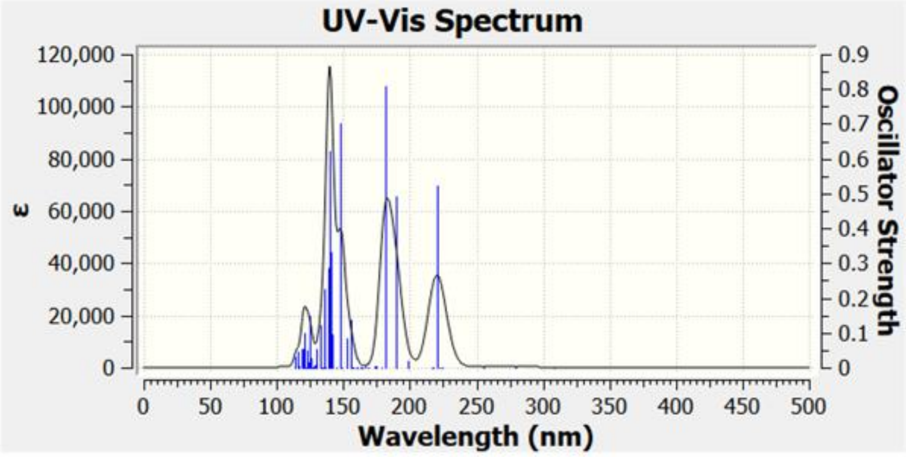

Figure S11: Theoretical extinctions (bottom) and ECD spectrum (top) of KKI18 (*Z,E*) calculated at the B3LYP/6-311+G(d,p) level of theory.

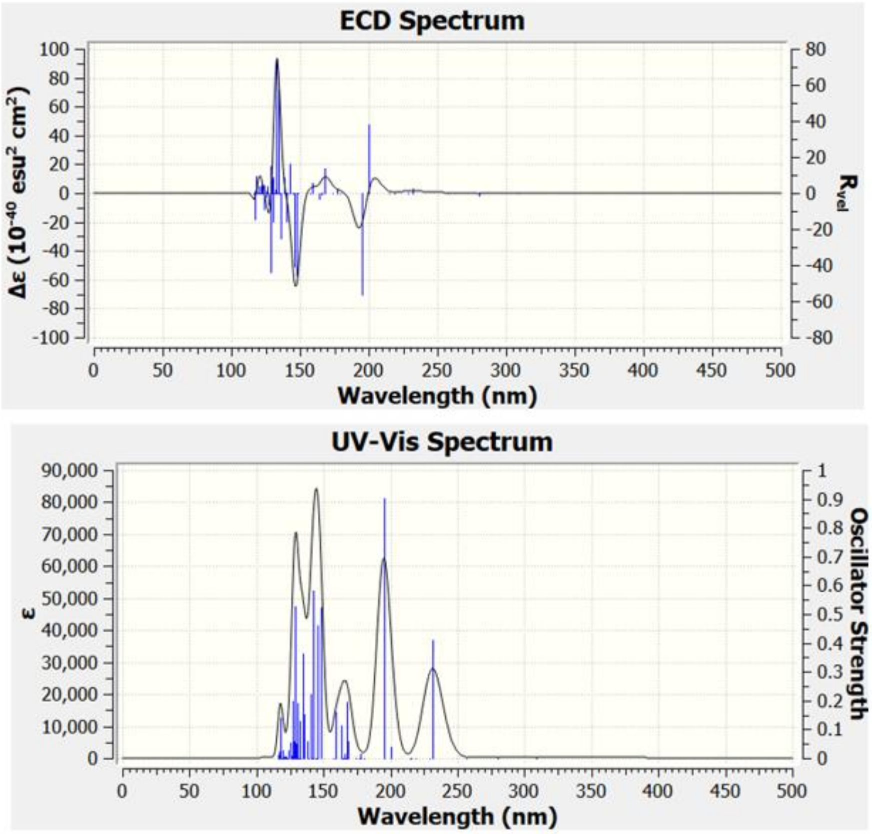

Figure S12: Theoretical extinctions (bottom) and ECD spectrum (top) of KKI18 (*Z,Z*) calculated at the B3LYP/6-311+G(d,p) level of theory.

Molecular orbitals calculation

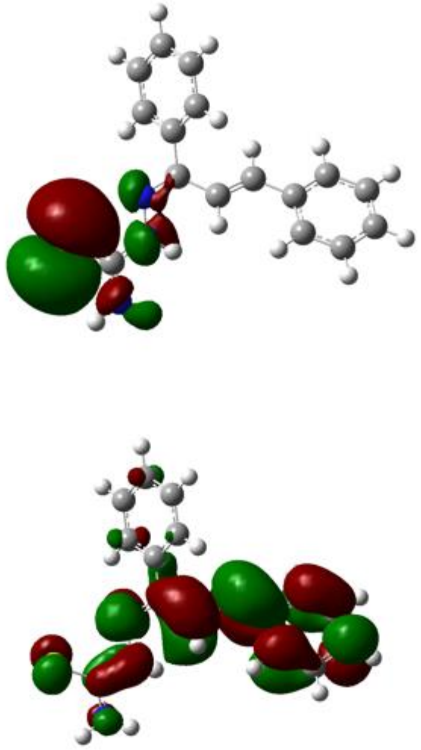

Figure S13: Orbitals in HOMO (top) and LUMO (bottom) in KKI15 (*E,E*) conformation.

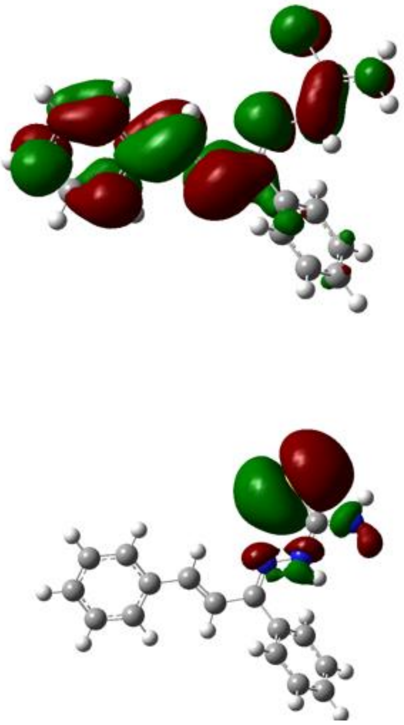

Figure S14: Orbitals in HOMO (bottom) and LUMO (top) in KKI15 (*E,Z*) conformation.

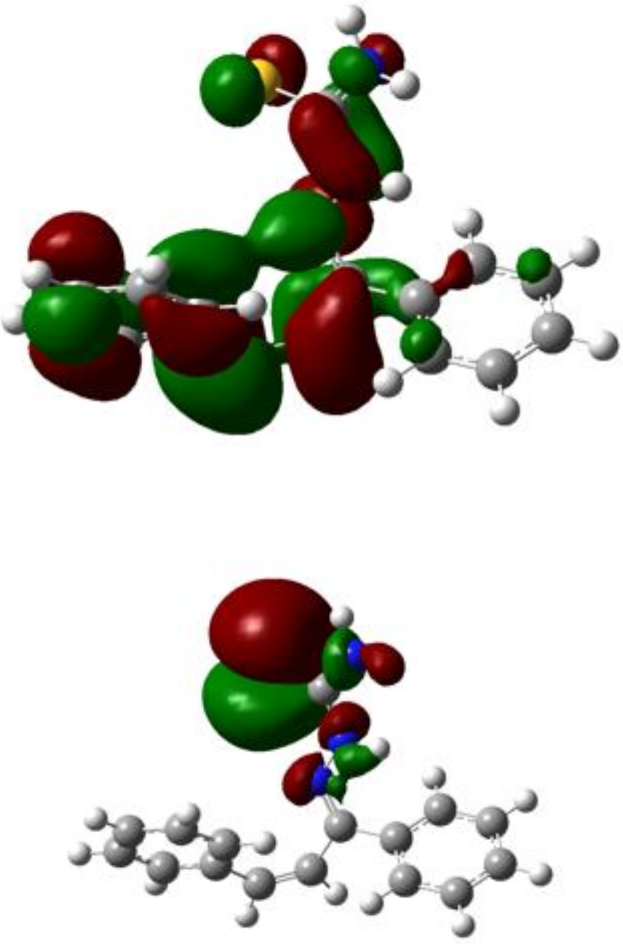

Figure S15: Orbitals in HOMO (bottom) and LUMO (top) in KKI15 (*Z,E*) conformation.

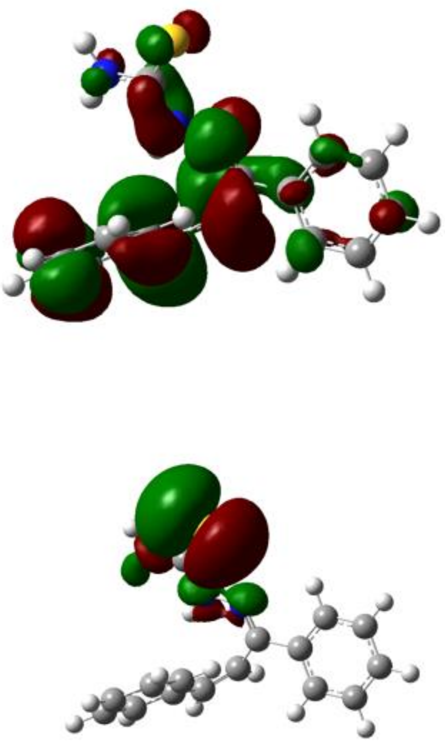

Figure S16: Orbitals in HOMO (bottom) and LUMO (top) in KKI15 (*Z,Z*) conformation.

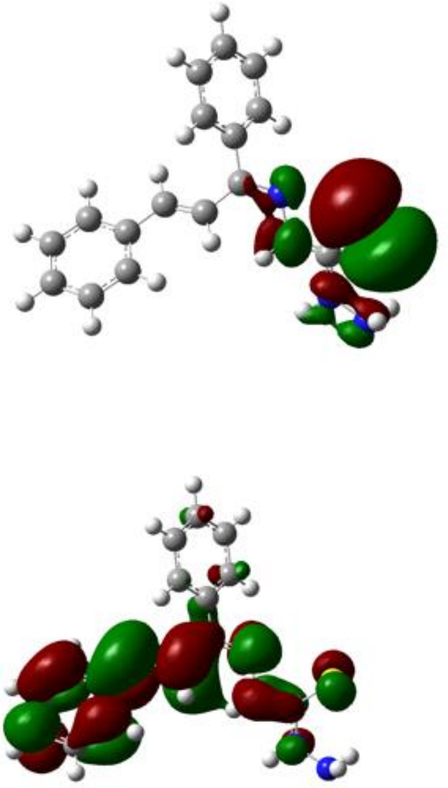

Figure S17: Orbitals in HOMO (bottom) and LUMO (top) in KKI18 (*E,E)* conformation.

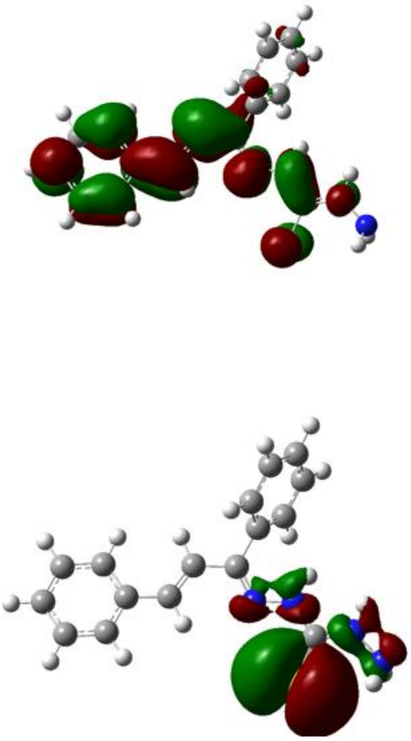

Figure S18: Orbitals in HOMO (bottom) and LUMO (top) in KKI18 (*E,Z)* conformation.

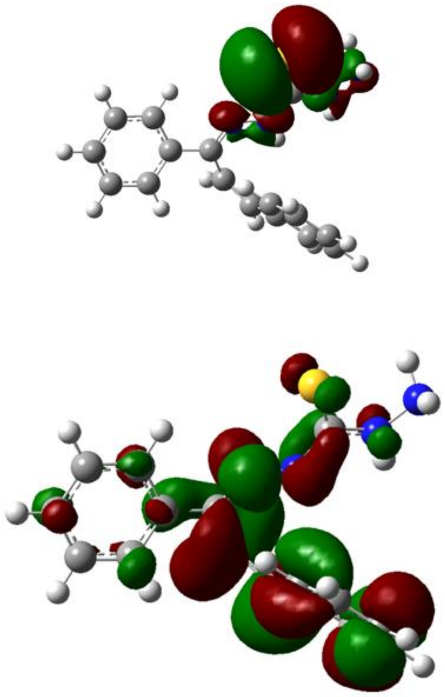

Figure S19: Orbitals in HOMO (top) and LUMO (bottom) in KKI18 (*Z,E)* conformation.

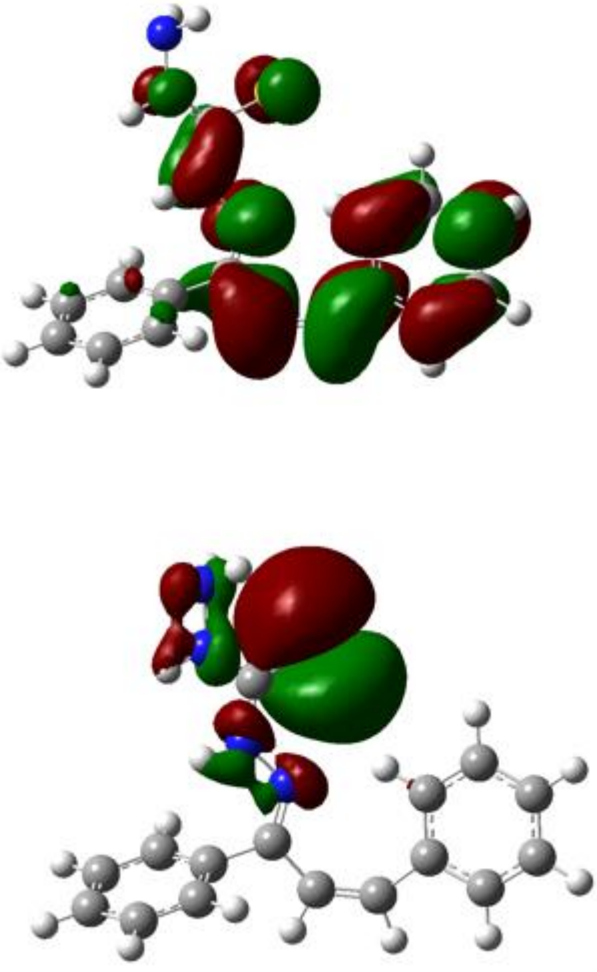

Figure S20: Orbitals in HOMO (bottom) and LUMO (top) in KKI18 (*Z,Z)* conformation.

Table S3: UV-vis absorption spectra and ECD spectra. Main λmax (nm) and oscillator strengths in parenthesis of the calculated structures

**Conformation**	**UV-Vis**	
**KKI15**	**Triplet excitations**	**Singlet excitations**
E/E	695.77	234.53(0.9874) 204.67(0.3673) 180.45(0) 147.27(0) 127.97(0)
E/Z	685.33	234.74(0.7854) 197.54(0.9241) 142.21(0.6315) 131.19(0.431)
Z/E	709.15	224.76(0) 181.09(0.7656) 145.31(0) 120.81(0.101)
Z/Z	705.33	222.95(0) 188.93(0.6636) 141.13(0)
**KKI18**		
E/E	843.97/308.83	233.71(1.0126) 180.49(0) 146.86(0.7823) 127.15(0) 118.2(0.0198)
E/Z	795.80/308.91	233.75(0.8431) 196.33(0.9105) 142.24(0.6422) 129.86(0)
Z/E	819.65/309.16	222.92(0) 178.88(0) 144.49(0) 118.96(0.0517)
Z/Z	793.03/309.66	231.56(0.4121) 194.97(0.9014) 165.78(0.014) 140.4(0.223)
	**ECD**	
**KKI15**		
E/E	234.53(91.1629) 196.61(-42.8498) 119.04(-18.1054) 138.92(-80.2869)	
E/Z	234.74(57.4506) 201.45(-44.5427) 139.22(-72.9812) 131.19(171.5019) 123.82(-4.9979)	
Z/E	189.5(-104.56) 148.55(0) 136.89(0)	
Z/Z	222.95(0) 188.93(45.048) 147.119(0) 131.72(0)	
**KKI18**		
E/E	233.71(-103.7233) 195.45(47.0936) 146.86(-96.2542) 139.299(0) 119.11(11.2436)	
E/Z	233.75(-59.7823) 201.29(58.9464) 169.44(0) 143.66(0) 131.29(0) 122.98(0)	
Z/E	189.51(101.5477) 174.71(-0.4105) 148.69(0) 136.68(0)	
Z/Z	194.97(-56.2307) 168.44(0) 147.74(-45.6857) 133.33(0) 121.48(4.0126)	

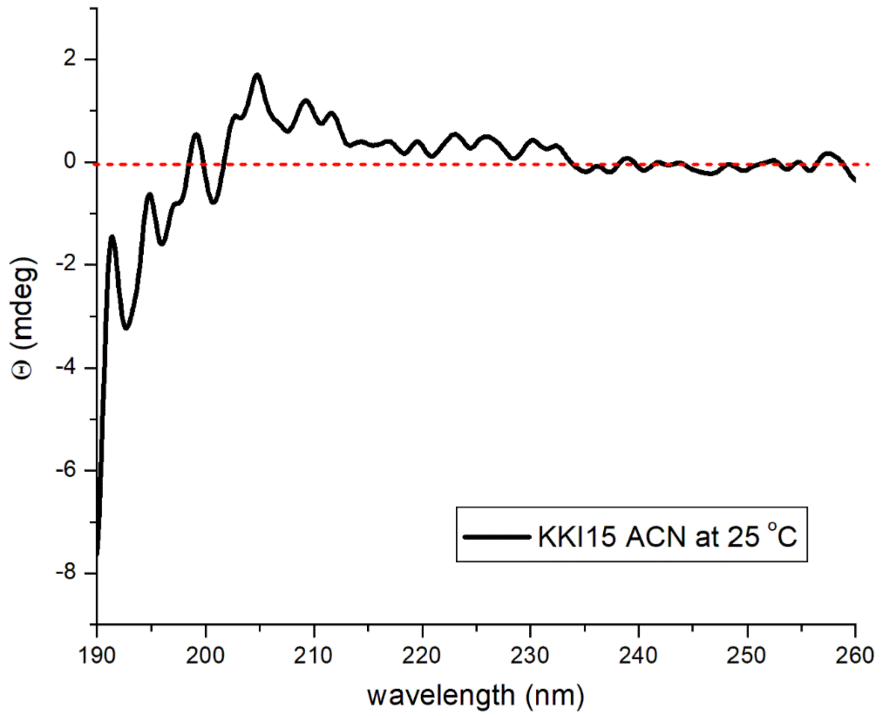

Figure S21: Circular Dichroism of KKI15.

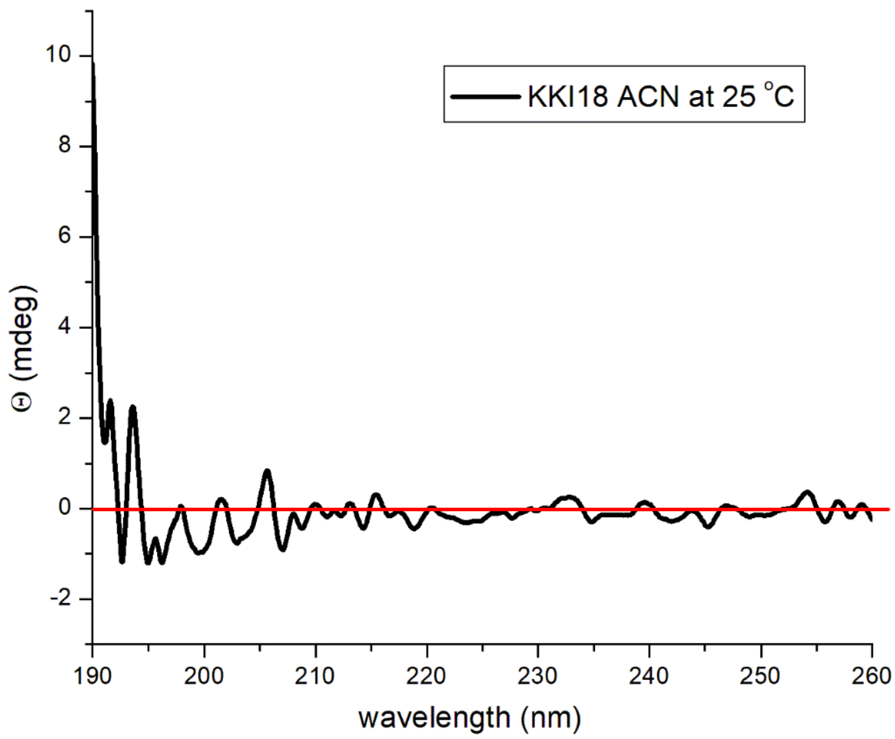

Figure S22: Circular Dichroism of KKI18.
